# Dynamical behavior of water wave phenomena for the 3D fractional WBBM equations using rational sine-Gordon expansion method

**DOI:** 10.1038/s41598-024-55215-1

**Published:** 2024-03-18

**Authors:** Abdulla-Al- Mamun, Chunhui Lu, Samsun Nahar Ananna, Md Mohi Uddin

**Affiliations:** 1 College of Hydrology and Water Resources, Hohai University, Nanjing, 210098 People’s Republic of China; 2 State Key Laboratory of Hydrology-Water Resources and Hydraulic Engineering, Hohai University, Nanjing, People’s Republic of China; 3 Department of Computer Science and Engineering, Northern University of Business and Technology Khulna, Khulna, 9100 Bangladesh; 4 School of Mathematics, Hohai University, Nanjing, 210098 People’s Republic of China; 5 College of Water Conservancy and Hydropower, Hohai University, Nanjing, 210098 People’s Republic of China

**Keywords:** Wazwaz-Benjamin-Bona-Mahony equation, The rational sine-Gordon expansion method, Exact solution, Soliton shape, Lump shape, Sine-Gordon equation, Hydrology, Mathematics and computing, Physics

## Abstract

To examine the dynamical behavior of travelling wave solutions of the water wave phenomenon for the family of 3D fractional Wazwaz-Benjamin-Bona-Mahony (WBBM) equations, this work employs the rational Sine-Gordon expansion (RSGE) approach based on the conformable fractional derivative. The method generalizes the well-known sine-Gordon expansion using the sine-Gordon equation as an auxiliary equation. In contrast to the conventional sine-Gordon expansion method, it takes a more general approach, a rational function rather than a polynomial one of the solutions of the auxiliary equation. The method described above is used to generate various solutions of the WBBM equations for hyperbolic functions, including soliton, singular soliton, multiple-soliton, kink, cusp, lump-kink, kink double-soliton, etc. The RSGE method contributes to our understanding of nonlinear phenomena, provides exact solutions to nonlinear equations, aids in studying solitons, advances mathematical techniques, and finds applications in various scientific and engineering disciplines. The answers are graphically shown in three-dimensional (3D) surface plots and contour plots using the MATLAB program. The resolutions of the equation, which have appropriate parameters, exhibit the absolute wave configurations in all screens. Furthermore, it can be inferred that the physical characteristics of the discovered solutions and their features may aid in our understanding of the propagation of shallow water waves in nonlinear dynamics.

## Introduction

Numerous issues in applied sciences, such as fluid dynamics, hydrodynamics, plasma physics, and quantum mechanics, may be modelled using ordinary and partial differential equations to characterize their physical characteristics under suitable conditions. Ordinary differential equations (ODEs) are more accessible to solve analytically, but partial differential equations (PDEs), especially nonlinear equations, are more challenging. PDEs typically convert to ODEs when they seek explicit solutions using the Ansatz (direct) and Symmetry techniques. To verify correctness and compare numerical systems, exact solutions are helpful.

To create a flow in a domain, air must be replaced by water in soils (and foams) or vice versa in fluid recovery operations. Newton's law of viscosity, which stipulates that the shear stress between adjacent fluid layers is proportional to the velocity gradient between the two layers, is not followed by non-Newtonian fluids. Newtonian fluids have a constant viscosity regardless of the force applied. Conversely, non-Newtonian fluids can experience variations in density due to various factors, including shear rate or stress. Because of their varied behavior, non-Newtonian fluids cannot all be described by a single model. Several models relate their viscosity to pressure or shear rate^1–5^. Both systems have equivalent principles regulating fluid flow. However, depending on the medium under consideration, these laws may be represented differently or use different terminology. Although flow in soil and flow in foam research disciplines are concerned with comparable physical laws^6,7^, communication between them has been hampered by a lack of ordinary language. A frequent and intriguing example of travelling waves in nature is water waves. The water surface oscillates up and down as a travelling wave travels across it, generating wave patterns that move over the surface. The characteristics of water waves, such as their wavelength, frequency, speed, and amplitude, may be used to define how they behave. The wave equation, a partial differential equation that describes the correlation between wave motion, time, and space, controls the dynamics of water waves. The one-dimensional linear shallow water wave equation, often known as the Korteweg-de Vries equation (KdV)^8^, is the traditional wave equation for small amplitude waves in shallow water. It defines waves that may move without altering form and have a single wave profile. Waves in water often disperse, which means they move at varying rates depending on their wavelength. Shorter waves with higher frequencies move more slowly than longer waves with lower frequencies.

**Figure 1:**
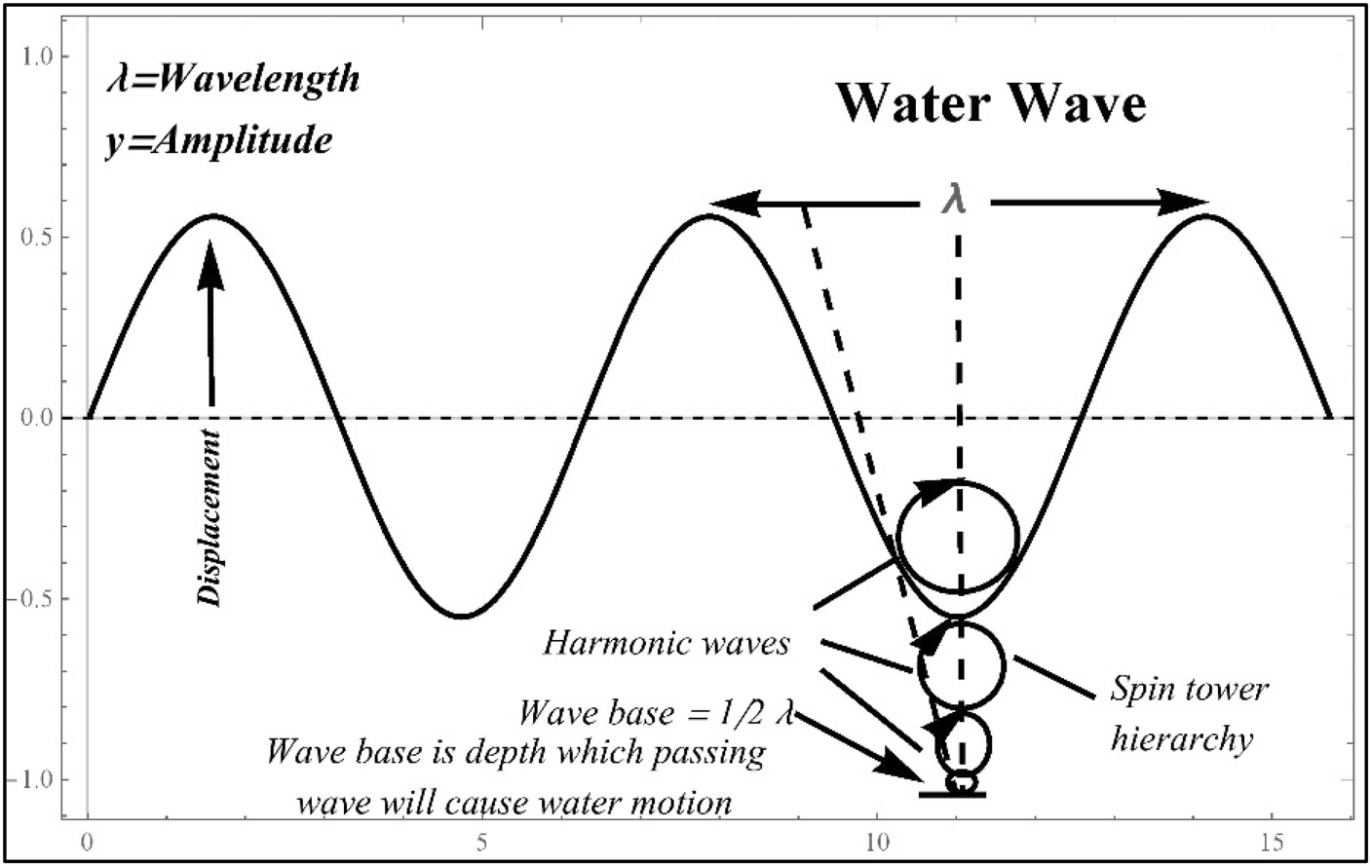
Dynamics of water waves.

The interactions between the waves and the surface tension and depth of the water cause this dispersion. The dynamics of water waves can become nonlinear for huge amplitude waves over long distances^9^. Due to nonlinear wave dynamics, complex patterns like solitons and rogue waves can arise. Waves may grow steep and unstable as they go closer to shallow water, finally breaking into choppy whitecaps. Near shorelines, this phenomenon is pronounced. Figure 1 represents the dynamics of water waves. In general, water waves display a wide range of characteristics, making them an important topic of interest and research in fluid dynamics, oceanography, and other related ones. Numerous variables affect their dynamic behavior, including wave characteristics, water depth, and environmental interactions^10,11^.

A family of nonlinear partial differential equations called the Wazwaz-Benjamin-Bona-Mahony (WBBM) equations is used to simulate the behavior of water waves. They expand the Benjamin-Bona-Mahony (BBM) equation, which explains long waves in the context of weak dispersiveness and nonlinearity. Wave propagation non-locality and memory effects are taken into consideration in the WBBM equations through the use of fractional derivatives. The fractional Wazwaz-BBM (WBBM) equation is an extension proposed by Wazwaz to incorporate fractional calculus into the BBM equation. The specific form of the 3D fractional WBBM equations would depend on the dimensionality and the particular fractional derivative operator used. In 2017, Wazwaz^12^ proclaimed a distinctive structure for the 3D architecture of the upgraded BBM equations, a framework, and an overview of differing thoughts used in the literature^8,13–17^. The subsequent are the new equations, frequently known as the WBBM equations:

1 $${{\text{u}}}_{{\text{x}}}+{{\text{u}}}^{2}{{\text{u}}}_{{\text{y}}}+{{\text{u}}}_{{\text{t}}}-{{\text{u}}}_{{\text{xzt}}}=0,$$

2 $${{\text{u}}}_{{\text{z}}}+{{\text{u}}}^{2}{{\text{u}}}_{{\text{x}}}+{{\text{u}}}_{{\text{t}}}-{{\text{u}}}_{{\text{xyt}}}=0,$$

3 $${{\text{u}}}_{{\text{y}}}+{{\text{u}}}^{2}{{\text{u}}}_{{\text{z}}}+{{\text{u}}}_{{\text{t}}}-{{\text{u}}}_{{\text{xxt}}}=0.$$

These particular fractional equations are frequently encountered in investigating complex phenomena in various scientific disciplines, including fluid dynamics, nonlinear waves, and other areas where long-range interactions or fractal-like behavior are present. Therefore, those, as mentioned earlier, recently discovered equations will be explored in this work, assuming that challenges in higher dimensions have more incredible applications in authentic situations and that solutions assist in adopting the extended belated physics models.

The main goal of this work is to directly apply the RSGE method to the dynamical analysis of new 3D fractional WBBM equations. There are several benefits when comparing our strategy to the other approaches. Simply put, it employs a more structured technique and more steps to generate an algebraic system. It also automatically creates kink and singular soliton solutions^17–19^. The principal important methodology of this method is too explicit the exact solutions of FNLEEs that satisfy the Nonlinear ODE of the form, $U\left( \psi \right) = \sum\nolimits_{{i = 1}}^{N} {\tanh ^{{i - 1}} \psi \left( {a_{i} \;{\text{sech}}\;\psi + c_{i} \tanh \psi + a_{0} } \right)} /\sum\nolimits_{{i = 1}}^{N} {\tanh ^{{i - 1}} \psi \left( {b_{i} \;{\text{sech}}\;\psi + d_{i} \tanh \psi + b_{0} } \right)}$. Our method provides a more direct and concise approach to the exact travelling wave solution than the other existing systems. Some authors used this RSGE technique to determine the exact solution to multiple NLEEs in the deferential sense of derivative, such as Jumarie's modified Riemann–Liouville derivatives, conformable derivatives, and Kerr law nonlinearity. Nevertheless, no adequate studies utilizing this method have been conducted on our suggested WBBM equation. Here, the recently found exact solution of the WBBM equations is more accurate, efficient, and versatile enough to be used in many treatments in mathematical physics, engineering, and wave analysis. Thus, we can state that our proposed research is innovative in the sense of conformable derivatives as it employs the RSGE technique to dynamically analyze recently introduced WBBM equations. We presented the results using the mathematical software Mathematica by choosing appropriate values for the employed parameters and then employing illustrations to simplify the physical interpretation suitably.

Recent years have significantly increased interest in nonlinear fractional differential equations. The change in fractional calculus theory and the use of such shapes in various fields, including physics, engineering, and biology, depend greatly^20,21^. To discriminate between different sorts of nonlinear circumstances in applied research and engineering, travelling wave solutions of nonlinear partial differential equations must be studied. Heat flow, shallow water waves, wave propagation, optical fibres, plasma physics, fluid mechanics, biology, electricity, chemical kinematics, and quantum theory are only a few examples of the numerous nonlinear wave techniques that have been employed in the past to illustrate different physics issues^22–24^. Thus, to investigate these substances, scores of effective strategies have been recommended in the circulated works by scholars, namely the improved modified extended tanh-function method^17^, the (G′/G,1/G)-expansion technique^15,25^, the modified extended tanh-function method^8,26,27^, the (Gʹ/G^2^)-expansion technique^16,28^, the advanced exp(− ∅(ξ))-expansion method^29–31^, the tanh-coth method^32^, the variational iteration method^33–35^, the method of characteristics^36^, the exp function method^37^, the sine-Gordon expansion method^18^, Wang's Bäcklund transformation-based method^38^, the new auxiliary equation method^19^, the Hirota bilinear method^39–41^, Soret and Dufour effects^42^, Variational method^43^, Deep Learning approach^44^, Dixon resultant method^45^, the three-dimensional molecular structure model^46^, the Kudryashov method^47^, etc.

The layout of the article is as follows: the critical stages of the chosen approaches are provided in Section "Introduction". In the section under "Rational sine-Gordon expansion method", we apply the most recent version of the Rational sine-Gordon expansion method to our chosen models, the 3D fractional WBBM equations. Results analysis is offered in the section under "Result and discussion". The "Conclusion" section contains the primary synopsis of our new work.

## Conformable fractional derivative

Some researchers^48–55^ have utilized the above description of fractional derivatives and several of their attributes to investigate exact solutions for numerous fractional differential equations. Significant research on conformable derivatives is being conducted and should be emphasized here^54^. Recently, the conformable derivative's geometric and physical representations have been made more apparent^55^. To describe the physical ecosphere, they presented the general conformable derivative, a family of innovative fractional derivatives having geometrical and physical implications. It is the thorough process for the conformable derivative that Khalil^56^ proposed. This section briefly discussesonformable fractional derivatives and their properties^56–58^.

**Definition 1**.^56^ Based on the independent variable t, the conformable derivative of order $\upgamma$ is defined as

4 $${{\text{D}}}_{{\text{t}}}^{\upgamma }\left({\text{z}}\left({\text{t}}\right)\right)=\underset{\uprho \to 0}{{\text{lim}}}\frac{{\text{z}}\left({\text{t}}+\uprho {{\text{t}}}^{1-\upgamma }\right)-{\text{z}}({\text{t}})}{\uprho },\quad {\text{t}}>0,\quad \upgamma \in \left(\text{0,1}\right].$$

for persistence ${\text{z}}={\text{z}}\left({\text{t}}\right):\left[0,\infty \right)\to {\mathbb{R}}$. This well-known fractional derivative is achieved by satisfying some known necessary conditions. If y is $\upgamma$-differential in some (0, ρ), ρ > 0, and then $\underset{{\text{t}}\to 0+}{{\text{lim}}}{{\text{D}}}_{{\text{t}}}^{\upgamma }\left({\text{y}}\left({\text{t}}\right)\right)$ exists, then

$${{\text{D}}}_{{\text{t}}}^{\upgamma }\left({\text{z}}\left(0\right)\right)=\underset{{\text{t}}\to 0+}{{\text{lim}}}{{\text{D}}}_{{\text{t}}}^{\upgamma }\left({\text{z}}\left({\text{t}}\right)\right).$$

**Theorem 1**.^57^ Suppose the derivative order $\upgamma \in (0, 1]$, and undertake that for all positive values of t, $\upmu =\upmu ({\text{t}})$ and $\upsigma =\upsigma ({\text{t}})$ are $\upgamma$-differentiable. Then,

- $${{\text{D}}}_{{\text{t}}}^{\upgamma }\left({{\text{m}}}_{1}\upmu +{{\text{m}}}_{2}\upsigma \right)={{\text{m}}}_{1}{{\text{D}}}_{{\text{t}}}^{\upgamma }\left(\upmu \right)+{{\text{m}}}_{2}{{\text{D}}}_{{\text{t}}}^{\upgamma }\left(\upsigma \right),$$
- $${{\text{D}}}_{{\text{t}}}^{\upgamma }\left({{\text{t}}}^{{\text{p}}}\right)={{\text{pt}}}^{{\text{p}}-\upgamma }, \forall {\text{p}}\in {\mathbb{R}},$$
- $${{\text{D}}}_{{\text{t}}}^{\upgamma }\left(\upbeta \right)=0, \forall {\text{u}}\left({\text{t}}\right)=\upbeta ,$$
- $${{\text{D}}}_{{\text{t}}}^{\upgamma }\left({\mu \sigma }\right)=\upmu {{\text{D}}}_{{\text{t}}}^{\upgamma }\left(\upsigma \right)+\upsigma {{\text{D}}}_{{\text{t}}}^{\upgamma }\left(\upmu \right),$$
- $${{\text{D}}}_{{\text{t}}}^{\upgamma }\left(\frac{\upmu }{\upsigma }\right)=\frac{{{\text{fD}}}_{{\text{t}}}^{\upgamma }\left(\upmu \right)-\upmu {{\text{D}}}_{{\text{t}}}^{\upgamma }\left(\upsigma \right)}{{\upsigma }^{2}},$$
- $${{\text{D}}}_{{\text{t}}}^{\upgamma }\left(\upmu \right)\left({\text{t}}\right)={{\text{t}}}^{1-\upgamma }\frac{{\text{d}}\upmu }{{\text{dt}}}.$$

for all ${{\text{c}}}_{1},{{\text{c}}}_{2}\in {\mathbb{R}}$. The conformable differential operator follows some vital essential stuff similar to the chain rule, Laplace transforms, and Taylor series expansion^58^.

**Theorem 2**. Consider $\upmu =\upmu ({\text{t}})$ be a $\upgamma$ conformable differentiable function and undertake that ${\text{f}}$ is differentiable and precise in various ${\text{g}}$. Then,

5 $${{\text{D}}}_{{\text{t}}}^{\upgamma }\left(\upmu \circ\upsigma \right)\left({\text{t}}\right)={{\text{t}}}^{1-\upgamma }{\upsigma }^{\prime}\left({\text{t}}\right){\upmu }^{\prime}\left(\upsigma \left({\text{t}}\right)\right),$$

The conformable fractional derivative has two benefits over conventional fractional derivatives. First, the definition of the conformable fractional derivative is likely accurate. It satisfies most conditions for the classical integral derivative, such as linearity, Rolle's theorem, mean value theorem, product rule, quotient rule, power rule, and chain rule. Second, for modelling various physical problems, the conformable derivative is far more helpful than the Riemann–Liouville or Caputo fractional derivatives. This is because, compared to the latter two, the differential equations related to the conformable fractional derivative are more accessible to solve theoretically. Several academics have previously employed conformable fractional derivatives in various fields and created numerous matching strategies.

## Rational sine-Gordon expansion method

The consistent fractional form $u\left(x,t\right)=U(\uppsi )$ with $\uppsi =a\left(x-\frac{v{t}^{\gamma }}{\gamma }\right)$ The unadventurous wave renovation^18,59,60^ decreases the fractional Sine-Gordon equation in one dimension of the form

6 $$\frac{{\partial }^{2}u}{\partial {x}^{2}}-{D}_{t}^{2\alpha }u={m}^{2}{\text{sin}}u,\;m\;is\;constant.$$

To the ODE

7 $$\frac{{d}^{2}U}{d{\uppsi }^{2}}=\frac{{m}^{2}}{{a}^{2}\left(1-{v}^{2}\right)}{\text{sin}}\;U,$$

where $v$ indicates the velocity of the travelling wave illustrated in the transform. Reference^32^ Some simplifications lead

8 $${\left(\frac{d\left(\frac{U}{2}\right)}{d\uppsi }\right)}^{2}=\frac{{m}^{2}}{{a}^{2}\left(1-{v}^{2}\right)}{{\text{sin}}}^{2}\frac{U}{2}+C,$$

where C is an integrating constant and is supposed to be zero for simplicity. Let $w\left(\uppsi \right)=\frac{U\left(\uppsi \right)}{2}$ and ${b}^{2}=\frac{{m}^{2}}{{a}^{2}\left(1-{v}^{2}\right)}$. Then Eq. (8) is converted to

9 $$\frac{d\left(w\right)}{d\uppsi }=b\;{\text{sin}}\;w.$$

Set $b=1$ in Eq. (9). Then Eq. (9) yields two significant relations.

10 $${\text{sin}}\;w(\uppsi )={\left.\frac{2d{e}^{\uppsi }}{{d}^{2}{e}^{2\uppsi }+1}\right|}_{d=1}=\text{sech}\;{\psi },$$

or

11 $${\text{cos}}\;w\left(\uppsi \right)={\left.\frac{{d}^{2}{e}^{2\uppsi }-1}{{d}^{2}{e}^{2\uppsi }+1}\right|}_{d=1}=\text{tanh}\;{\psi },$$

where $d$ is a nonzero integrating constant. Then, the fractional PDE of the form

12 $$P\left(u, {D}_{t}^{\gamma }u, {u}_{x}, {D}_{tt}^{2\gamma }, {u}_{xx}, \dots \right)=0,$$

can be reduced to an ODE

13 $$\widetilde{P}=\left(U,{U}^{\prime},{U}^{{\prime\prime}},\dots \right)=0,$$

by using an equivalent wave transform $u\left(x,t\right)=U(\uppsi )$ where the transform variable $\uppsi$ is specified as $a\left(x-\frac{v{t}^{\gamma }}{\alpha }\right)$. Then, the expected solution (Eq. 13) of the form

14 $$U\left(\uppsi \right)={A}_{0}+\sum_{i=1}^{s}{{\text{tanh}}}^{i-1}\left(\uppsi \right)\left({B}_{i}\;\text{sech}\;{\psi }+{A}_{i}\;\text{tanh}\;{\psi }\right),$$

can be written as

15 $$U\left(w\right)={A}_{0}+\sum_{i=1}^{s}{{\text{cos}}}^{i-1}\left(w\right)\left({B}_{i}\;{\text{sin}}\;w+{A}_{i}\;{\text{cos}}\;w\right).$$

use Eqs. (10) and (11), Eq. (9) is a bivariate polynomial function in $\text{tanh}{\psi }$ and $\text{sech}{\psi }$, as is evident. Due to the relationships ${{\text{tanh}}}^{2}\uppsi +{{\text{sech}}}^{2}\uppsi =1$, it is essential to note that this polynomial must be linear in one of these auxiliary functions. In this case, $\text{sech}{\psi }$. We can now see that a subset of rational functions comprises polynomial functions. As a result, the latter is often far superior to the former in tasks like interpolation or approximating functions^61^. It is simple to assume that the same will hold while attempting to solve nonlinear evolution equations. The concept of rational expansion has been utilized in the literature before, but only in the context of one auxiliary function^61–63^. In this study, we propose expanding this concept to two additional tasks.

16 $$U\left(\uppsi \right)=\frac{\sum_{i=1}^{N}{{\text{tanh}}}^{i-1}\uppsi \left({a}_{i}\;\text{sech}\;{\psi }+{c}_{i}\;{{\text{tanh}}}\;{\psi }+{a}_{0}\right)}{\sum_{i=1}^{N}{{\text{tanh}}}^{i-1}\uppsi \left({b}_{i}\;\text{sech}\;{\psi }+{d}_{i}{{{\text{tanh}}}}\;{\psi }+{b}_{0}\right)},$$

in place of Eq. (14), which can also be written as

17 $$U\left({\text{w}}\right)=\frac{\sum_{i=1}^{N}{{\text{cos}}}^{i-1}{\text{w}}\left({a}_{i}\;{\text{sinw}}+{c}_{i}\;{\text{cosw}}+{a}_{0}\right)}{\sum_{i=1}^{N}{{\text{cos}}}^{i-1}\;{\text{w}}\;\left({b}_{i}\;{\text{sinw}}+{d}_{i}\;{\text{cosw}}+{b}_{0}\right)},$$

owing to Eqs. (16, 17). Setting up index limits with a uniform balance of the conditions in Eq. (13) is the first step in the procedure. The projected solution (Eq. 16), engaging in Eq. (13), is replaced, and the coefficient of powers of sin and cos is assumed to be zero. Next, an explanation of the ensuing algebraic system is provided for the coefficients ${a}_{0},{a}_{1},{b}_{0},{b}_{1},\dots$. If there are any answers, they are put together using Eqs. (10, 11) and $\uppsi$.

## Application of the RSGE method

### The 1st WBBM equation

Consider the 3D fractional WBBM equation as follows:

18 $${{\text{D}}}_{{\text{t}}}^{\upgamma }{\text{u}}+{{\text{D}}}_{{\text{x}}}^{\upgamma }{\text{u}}+{{\text{D}}}_{{\text{y}}}^{\upgamma }{{\text{u}}}^{3}-{{\text{D}}}_{{\text{xzt}}}^{3\upgamma }{\text{u}}=0.$$

Utilizing the subsequent wave transformation

$$\text{u }\left({\text{x}},{\text{y}},{\text{z}},{\text{t}}\right)=\text{U }\left(\uppsi \right),\text{ where}\;{\psi }=\text{p }\frac{{{\text{x}}}^{\upgamma }}{\upgamma }+\text{q }\frac{{{\text{y}}}^{\upgamma }}{\upgamma }+\text{r }\frac{{{\text{z}}}^{\upgamma }}{\upgamma }-\text{s }\frac{{{\text{t}}}^{\upgamma }}{\upgamma }.$$

on Eq. (18), we get

19 $$\left(-{\text{s}}+{\text{p}}\right) {{\text{U}}}^{\prime}+\text{q }{\left({{\text{U}}}^{3}\right)}^{\prime}+{\text{p r s U}}^{{\prime\prime}}=0.$$

Integrating Eq. (19) with respect to $\psi$, we get

$$\left(-{\text{s}}+{\text{p}}\right)\text{ U}+{\text{q U}}^{3}+{\text{p r s U}}^{\prime\prime}+{{\text{c}}}_{1}=0,$$

where ${c}_{1}$ is an integrating constant. We set ${c}_{1}=0$ for simplicity, we get,

20 $$\left(-{\text{s}}+{\text{p}}\right)\text{ U}+{\text{q U}}^{3}+{\text{p r s U}}^{\prime\prime}=0.$$

With the integrity of homogeneous evaluating in Eq. (20), we find that $N=1$. Thus, our endorsed technique allows us to use the supplementary solution of the form:

21 $$U\left(w\right)=\frac{{a}_{0} + {a}_{1}{\text{sin}}w + {c}_{1}{\text{cos}} w}{{b}_{0} + {b}_{1} {\text{sin}}w + {d}_{1} {\text{cos}}w},$$

Now employing the value of $U,U^{\prime\prime}\;{\text{and}}\;U^{3}$ in Eq. (20) as well as comparing the coefficients of like power of $\upgamma$ from the above equation, we get the SAE as follows:

22 $$\begin{array}{l}q{a}_{0}^{3}+3q{a}_{0}{a}_{1}^{2}+p{a}_{0}{b}_{0}^{2}-s{a}_{0}{b}_{0}^{2}+2p{a}_{1}{b}_{0}{b}_{1}-2s{a}_{1}{b}_{0}{b}_{1}-prs{a}_{1}{b}_{0}{b}_{1}+p{a}_{0}{b}_{1}^{2}-s{a}_{0}{b}_{1}^{2}\\ -prs{a}_{0}{b}_{1}^{2}-2prs{b}_{0}{c}_{1}{d}_{1}+2prs{a}_{0}{d}_{1}^{2}=0,\\ 3q{a}_{0}^{2}{c}_{1}+3q{a}_{1}^{2}{c}_{1}+p{b}_{0}^{2}{c}_{1}-s{b}_{0}^{2}{c}_{1}-2prs{b}_{0}^{2}{c}_{1}+p{b}_{1}^{2}{c}_{1}-s{b}_{1}^{2}{c}_{1}+prs{b}_{1}^{2}{c}_{1}+2p{a}_{0}{b}_{0}{d}_{1}\\ -2s{a}_{0}{b}_{0}{d}_{1}+prs{a}_{0}{b}_{0}{d}_{1}+2p{a}_{1}{b}_{1}{d}_{1}-2s{a}_{1}{b}_{1}{d}_{1}-prs{a}_{1}{b}_{1}{d}_{1}=0,\\ -3q{a}_{0}{a}_{1}^{2}-2p{a}_{1}{b}_{0}{b}_{1}+2s{a}_{1}{b}_{0}{b}_{1}+prs{a}_{1}{b}_{0}{b}_{1}-p{a}_{0}{b}_{1}^{2}+s{a}_{0}{b}_{1}^{2}+3prs{a}_{0}{b}_{1}^{2}+3q{a}_{0}{c}_{1}^{2}\\ +2p{b}_{0}{c}_{1}{d}_{1}-2s{b}_{0}{c}_{1}{d}_{1}+2prs{b}_{0}{c}_{1}{d}_{1}+p{a}_{0}{d}_{1}^{2}-s{a}_{0}{d}_{1}^{2}-3prs{a}_{0}{d}_{1}^{2}=0,\\ -3q{a}_{1}^{2}{c}_{1}+2prs{b}_{0}^{2}{c}_{1}-p{b}_{1}^{2}{c}_{1}+s{b}_{1}^{2}{c}_{1}-prs{b}_{1}^{2}{c}_{1}+q{c}_{1}^{3}-prs{a}_{0}{b}_{0}{d}_{1}-2p{a}_{1}{b}_{1}{d}_{1}\\ +2s{a}_{1}{b}_{1}{d}_{1}+prs{a}_{1}{b}_{1}{d}_{1}+p{c}_{1}{d}_{1}^{2}-s{c}_{1}{d}_{1}^{2}=0,\\ -2prs{a}_{0}{b}_{1}^{2}+prs{a}_{0}{d}_{1}^{2}=0,\\ q{a}_{0}^{3}+3q{a}_{0}{a}_{1}^{2}+p{a}_{0}{b}_{0}^{2}-s{a}_{0}{b}_{0}^{2}+2p{a}_{1}{b}_{0}{b}_{1}-2s{a}_{1}{b}_{0}{b}_{1}-prs{a}_{1}{b}_{0}{b}_{1}+p{a}_{0}{b}_{1}^{2}-s{a}_{0}{b}_{1}^{2}\\ -prs{a}_{0}{b}_{1}^{2}-2prs{b}_{0}{c}_{1}{d}_{1}+2prs{a}_{0}{d}_{1}^{2}=0,\\ 3q{a}_{0}^{2}{a}_{1}+q{a}_{1}^{3}+p{a}_{1}{b}_{0}^{2}-s{a}_{1}{b}_{0}^{2}-prs{a}_{1}{b}_{0}^{2}+2p{a}_{0}{b}_{0}{b}_{1}-2s{a}_{0}{b}_{0}{b}_{1}-prs{a}_{0}{b}_{0}{b}_{1}+p{a}_{1}{b}_{1}^{2}\\ -s{a}_{1}{b}_{1}^{2}-2prs{b}_{1}{c}_{1}{d}_{1}+2prs{a}_{1}{d}_{1}^{2}=0,\\ 6q{a}_{0}{a}_{1}{c}_{1}+2p{b}_{0}{b}_{1}{c}_{1}-2s{b}_{0}{b}_{1}{c}_{1}-prs{b}_{0}{b}_{1}{c}_{1}+2p{a}_{1}{b}_{0}{d}_{1}-2s{a}_{1}{b}_{0}{d}_{1}+2prs{a}_{1}{b}_{0}{d}_{1}\\ +2p{a}_{0}{b}_{1}{d}_{1}-2s{a}_{0}{b}_{1}{d}_{1}-3prs{a}_{0}{b}_{1}{d}_{1}=0,\\ -q{a}_{1}^{3}+2prs{a}_{1}{b}_{0}^{2}-p{a}_{1}{b}_{1}^{2}+s{a}_{1}{b}_{1}^{2}+3q{a}_{1}{c}_{1}^{2}+2p{b}_{1}{c}_{1}{d}_{1}-2s{b}_{1}{c}_{1}{d}_{1}+prs{b}_{1}{c}_{1}{d}_{1}+p{a}_{1}{d}_{1}^{2}\\ -s{a}_{1}{d}_{1}^{2}-prs{a}_{1}{d}_{1}^{2}=0,\\ 2prs{a}_{0}{b}_{1}{d}_{1}=0.\end{array}$$

Explaining the SAE, we acquire the subsequent solution sets,

$$p=-\frac{2s}{-2+rs}, \quad {a}_{0}=0, \quad {a}_{1}=\pm \frac{i\sqrt{r}s\sqrt{-{b}_{0}^{2}+{b}_{1}^{2}}}{\sqrt{2q-qrs}}, \quad {c}_{1}=\pm \frac{\sqrt{r}s{b}_{0}}{\sqrt{-2q+qrs}}, \quad {d}_{1}=0.$$

$$p=\frac{s}{1+rs}, \quad {a}_{0}=0, \quad {a}_{1}=\pm \frac{\sqrt{2}\sqrt{r{s}^{2}{b}_{0}^{2}-r{s}^{2}{d}_{1}^{2}}}{\sqrt{q+qrs}}, \quad {b}_{1}=0, \quad {c}_{1}=0.$$

$$p=-\frac{2s}{-2+rs}, \quad {a}_{0}=0, \quad {a}_{1}=\pm \frac{\sqrt{r}s{b}_{0}}{\sqrt{2q-qrs}}, \quad {b}_{1}=0, \quad {c}_{1}=\pm \frac{\sqrt{r}s{b}_{0}}{\sqrt{-2q+qrs}}, \quad {d}_{1}=0.$$

$$p=-\frac{2s}{-2+rs}, \quad {a}_{0}=0, \quad {a}_{1}=0, \quad {b}_{0}=\pm {b}_{1}, \quad {c}_{1}=\pm \frac{\sqrt{r}s{b}_{1}}{\sqrt{-2q+qrs}}, \quad {d}_{1}=0.$$

$$p=\frac{s}{1+rs}, \quad {a}_{0}=0, \quad {a}_{1}=\pm \frac{\sqrt{2}\sqrt{r}s{d}_{1}}{\sqrt{-q-qrs}}, \quad {b}_{0}=0, \quad {b}_{1}=0, \quad {c}_{1}=0.$$

$$p=\frac{s}{1+rs}, \quad {a}_{0}=0, \quad {a}_{1}=\pm \frac{\sqrt{2}\sqrt{r}s{b}_{0}}{\sqrt{q+qrs}}, \quad {b}_{1}=0, \quad {c}_{1}=0, \quad {d}_{1}=0.$$

$$p=-\frac{s}{-1+2rs}, \quad {a}_{0}=0, \quad {a}_{1}=0, \quad {b}_{1}=0, \quad {c}_{1}=\pm \frac{\sqrt{2}\sqrt{r}s{b}_{0}}{\sqrt{-q+2qrs}}, \quad {d}_{1}=0.$$

Concerning these solution sets, we acquired the exact solutions of the equation

$${U}_{\text{1,2},\text{3,4}}\left(x,y,z,t\right)=\frac{\sqrt{r}s\left(\pm \frac{i{\text{Sech}}\left[\psi \right]\sqrt{-{b}_{0}^{2}+{b}_{1}^{2}}}{\sqrt{2q-qrs}}\pm \frac{{b}_{0}{\text{Tanh}}\left[\psi \right]}{\sqrt{q\left(-2+rs\right)}}\right)}{{b}_{0}+{\text{Sech}}\left[\psi \right]{b}_{1}};\uppsi =-\frac{s{t}^{\gamma }}{\gamma }-\frac{2s{x}^{\gamma }}{\left(-2+rs\right)\gamma }+\frac{q{y}^{\gamma }}{\gamma }+\frac{r{z}^{\gamma }}{\gamma }.$$

$${U}_{\text{5,6}}\left(x,y,z,t\right)=\pm \frac{\sqrt{2}{\text{Sech}}\left[\psi \right]\sqrt{r{s}^{2}\left({b}_{0}^{2}-{d}_{1}^{2}\right)}}{\sqrt{q+qrs}\left({b}_{0}+{d}_{1}{\text{Tanh}}\left[\psi \right]\right)};\uppsi =-\frac{s{t}^{\gamma }}{\gamma }+\frac{s{x}^{\gamma }}{\left(1+rs\right)\gamma }+\frac{q{y}^{\gamma }}{\gamma }+\frac{r{z}^{\gamma }}{\gamma }.$$

$${U}_{\text{7,8},\text{9,10}}\left(x,y,z,t\right)=\pm \frac{\sqrt{r}s{\text{Sech}}\left[\psi \right]\left(\sqrt{q\left(-2+rs\right)}\pm \sqrt{-q\left(-2+rs\right)}{\text{Sinh}}\left[\psi \right]\right)}{\sqrt{-{q}^{2}{\left(-2+rs\right)}^{2}}};\uppsi =-\frac{s{t}^{\gamma }}{\gamma }-\frac{2s{x}^{\gamma }}{\left(-2+rs\right)\gamma }+\frac{q{y}^{\gamma }}{\gamma }+\frac{r{z}^{\gamma }}{\gamma }.$$

$${U}_{\text{11,12}}\left(x,y,z,t\right)=\pm \frac{\sqrt{r}s{\text{Coth}}\left[\frac{\psi }{2}\right]}{\sqrt{q\left(-2+rs\right)}};\uppsi =-\frac{s{t}^{\gamma }}{\gamma }-\frac{2s{x}^{\gamma }}{\left(-2+rs\right)\gamma }+\frac{q{y}^{\gamma }}{\gamma }+\frac{r{z}^{\gamma }}{\gamma }.$$

$${U}_{\text{13,14}}\left(x,y,z,t\right)=\pm \frac{\sqrt{2}\sqrt{r}s{\text{Csch}}\left[\psi \right]}{\sqrt{-q\left(1+rs\right)}};\uppsi =-\frac{s{t}^{\gamma }}{\gamma }+\frac{s{x}^{\gamma }}{\left(1+rs\right)\gamma }+\frac{q{y}^{\gamma }}{\gamma }+\frac{r{z}^{\gamma }}{\gamma }.$$

$${U}_{\text{15,16}}\left(x,y,z,t\right)=\pm \frac{\sqrt{2}\sqrt{r}s{\text{Sech}}\left[\psi \right]}{\sqrt{q+qrs}};\uppsi =-\frac{s{t}^{\gamma }}{\gamma }+\frac{s{x}^{\gamma }}{\left(1+rs\right)\gamma }+\frac{q{y}^{\gamma }}{\gamma }+\frac{r{z}^{\gamma }}{\gamma }.$$

$${U}_{\text{17,18}}\left(x,y,z,t\right)=\pm \frac{\sqrt{2}\sqrt{r}s{\text{Tanh}}\left[\psi \right]}{\sqrt{q\left(-1+2rs\right)}};\uppsi =-\frac{s{t}^{\gamma }}{\gamma }-\frac{s{x}^{\gamma }}{\left(-1+2rs\right)\gamma }+\frac{q{y}^{\gamma }}{\gamma }+\frac{r{z}^{\gamma }}{\gamma }.$$

### The 2nd WBBM equation

The second WBBM equation has the following solution sets when the process mentioned above is followed:

$$p=\frac{2(r-s)}{qs}, \quad {a}_{0}=0, \quad {a}_{1}=\pm \frac{\sqrt{qs{b}_{0}^{2}-qs{b}_{1}^{2}}}{\sqrt{2}}, \quad {c}_{1}=\pm \frac{i\sqrt{q}\sqrt{s}{b}_{0}}{\sqrt{2}}, \quad {d}_{1}=0.$$

$$p=\frac{r-s}{2qs}, \quad {a}_{0}=-i\sqrt{2}\sqrt{q}\sqrt{s}{d}_{1}, \quad {a}_{1}=0, \quad {b}_{1}=0, \quad {c}_{1}=-i\sqrt{2}\sqrt{q}\sqrt{s}{b}_{0}.$$

$$p=\frac{r-s}{2qs}, \quad {a}_{0}=i\sqrt{2}\sqrt{q}\sqrt{s}{d}_{1}, \quad {a}_{1}=0, \quad {b}_{1}=0, \quad {c}_{1}=i\sqrt{2}\sqrt{q}\sqrt{s}{b}_{0}.$$

$$p=\frac{2(r-s)}{qs}, \quad {a}_{0}=-\frac{i\sqrt{q}\sqrt{s}{d}_{1}}{\sqrt{2}}, \quad {a}_{1}=-\frac{\sqrt{qs{b}_{0}^{2}-qs{d}_{1}^{2}}}{\sqrt{2}}, \quad {b}_{1}=0, \quad {c}_{1}=-\frac{i\sqrt{q}\sqrt{s}{b}_{0}}{\sqrt{2}}.$$

$$p=\frac{2(r-s)}{qs}, \quad {a}_{0}=\frac{i\sqrt{q}\sqrt{s}{d}_{1}}{\sqrt{2}}, \quad {a}_{1}=-\frac{\sqrt{qs{b}_{0}^{2}-qs{d}_{1}^{2}}}{\sqrt{2}}, \quad {b}_{1}=0, \quad {c}_{1}=\frac{i\sqrt{q}\sqrt{s}{b}_{0}}{\sqrt{2}}.$$

$$p=\frac{2(r-s)}{qs}, \quad {a}_{0}=-\frac{i\sqrt{q}\sqrt{s}{d}_{1}}{\sqrt{2}}, \quad {a}_{1}=\frac{\sqrt{qs{b}_{0}^{2}-qs{d}_{1}^{2}}}{\sqrt{2}}, \quad {b}_{1}=0, \quad {c}_{1}=-\frac{i\sqrt{q}\sqrt{s}{b}_{0}}{\sqrt{2}}.$$

$$p=\frac{2(r-s)}{qs}, \quad {a}_{0}=\frac{i\sqrt{q}\sqrt{s}{d}_{1}}{\sqrt{2}}, \quad {a}_{1}=\frac{\sqrt{qs{b}_{0}^{2}-qs{d}_{1}^{2}}}{\sqrt{2}}, \quad {b}_{1}=0, \quad {c}_{1}=\frac{i\sqrt{q}\sqrt{s}{b}_{0}}{\sqrt{2}}.$$

$$p=\frac{-r+s}{qs}, \quad {a}_{0}=0, \quad {a}_{1}=\pm \sqrt{2}\sqrt{qs{b}_{0}^{2}-qs{d}_{1}^{2}}, \quad {b}_{1}=0, \quad {c}_{1}=0.$$

$$p=\frac{2(r-s)}{qs}, \quad {a}_{0}=0, \quad {a}_{1}=0, \quad {b}_{0}=\pm {b}_{1}, \quad {c}_{1}=\pm \frac{i\sqrt{q}\sqrt{s}{b}_{1}}{\sqrt{2}}, \quad {d}_{1}=0.$$

$$p=\frac{r-s}{2qs}, \quad {a}_{0}=0, \quad {a}_{1}=0, \quad {b}_{1}=0, \quad {c}_{1}=\pm i\sqrt{2}\sqrt{q}\sqrt{s}{b}_{0}, \quad {d}_{1}=0.$$

$$p=\frac{2(r-s)}{qs}, \quad {a}_{0}=\pm \frac{i\sqrt{q}\sqrt{s}{d}_{1}}{\sqrt{2}}, \quad {a}_{1}=\pm \frac{i\sqrt{q}\sqrt{s}{d}_{1}}{\sqrt{2}}, \quad {b}_{0}=0, \quad {b}_{1}=0, \quad {c}_{1}=0.$$

$$p=\frac{2(r-s)}{qs}, \quad {a}_{0}=0, \quad {a}_{1}=\pm \frac{\sqrt{q}\sqrt{s}{b}_{0}}{\sqrt{2}}, \quad {b}_{1}=0, \quad {c}_{1}=\pm \frac{i\sqrt{q}\sqrt{s}{b}_{0}}{\sqrt{2}}, \quad {d}_{1}=0.$$

$$p=\frac{-r+s}{qs}, \quad {a}_{0}=0, \quad {a}_{1}=\pm i\sqrt{2}\sqrt{q}\sqrt{s}{d}_{1}, \quad {b}_{0}=0, \quad {b}_{1}=0, \quad {c}_{1}=0.$$

$$p=\frac{-r+s}{qs}, \quad {a}_{0}=0, \quad {a}_{1}=\pm \sqrt{2}\sqrt{q}\sqrt{s}{d}_{1}, \quad {b}_{0}=\pm \sqrt{2}{d}_{1}, \quad {b}_{1}=0, \quad {c}_{1}=0.$$

$$p=\frac{-r+s}{qs}, \quad {a}_{0}=0, \quad {a}_{1}=\pm \frac{i\sqrt{q}\sqrt{s}{d}_{1}}{\sqrt{2}}, \quad {b}_{0}=\pm \frac{1}{2}\sqrt{3}{d}_{1}, \quad {b}_{1}=0, \quad {c}_{1}=0.$$

$$p=\frac{-r+s}{qs}, \quad {a}_{0}=0, \quad {a}_{1}=\pm \sqrt{2}\sqrt{q}\sqrt{s}{b}_{0}, \quad {b}_{1}=0, \quad {c}_{1}=0, \quad {d}_{1}=0.$$

Concerning these solution sets, we acquired the exact solutions of the equation

$${U}_{\text{19,20,21,22}}\left(x,y,z,t\right)=\pm \frac{{\text{Sech}}\left[\psi \right]\left(i\sqrt{q}\sqrt{s}{\text{Sinh}}\left[\psi \right]{b}_{0}\pm \sqrt{qs\left({b}_{0}^{2}-{b}_{1}^{2}\right)}\right)}{\sqrt{2}({b}_{0}+{\text{Sech}}[\psi ]{b}_{1})};\uppsi =-\frac{s{t}^{\gamma }}{\gamma }+\frac{2\left(r-s\right){x}^{\gamma }}{qs\gamma }+\frac{q{y}^{\gamma }}{\gamma }+\frac{r{z}^{\gamma }}{\gamma }.$$

$${U}_{\text{23,24}}\left(x,y,z,t\right)=\pm \frac{i\sqrt{2}\sqrt{q}\sqrt{s}\left({d}_{1}+{b}_{0}{\text{Tanh}}\left[\psi \right]\right)}{{b}_{0}+{d}_{1}{\text{Tanh}}[\psi ]};\uppsi =-\frac{s{t}^{\gamma }}{\gamma }+\frac{(r-s){x}^{\gamma }}{2qs\gamma }+\frac{q{y}^{\gamma }}{\gamma }+\frac{r{z}^{\gamma }}{\gamma }.$$

$${U}_{\text{25,26,27,28}}\left(x,y,z,t\right)=\pm \frac{i\sqrt{q}\sqrt{s}{d}_{1}\pm {\text{Sech}}[\psi ]\sqrt{qs({b}_{0}^{2}-{d}_{1}^{2})}+i\sqrt{q}\sqrt{s}{b}_{0}{\text{Tanh}}[\psi ]}{\sqrt{2}({b}_{0}+{d}_{1}{\text{Tanh}}[\psi ])};\uppsi =-\frac{s{t}^{\gamma }}{\gamma }+\frac{2(r-s){x}^{\gamma }}{qs\gamma }+\frac{q{y}^{\gamma }}{\gamma }+\frac{r{z}^{\gamma }}{\gamma }.$$

$${U}_{\text{29,30}}\left(x,y,z,t\right)=\pm \frac{\sqrt{2}{\text{Sech}}[\psi ]\sqrt{qs\left({b}_{0}^{2}-{d}_{1}^{2}\right)}}{{b}_{0}+{d}_{1}{\text{Tanh}}[\psi ]};\uppsi =-\frac{s{t}^{\gamma }}{\gamma }+\frac{(-r+s){x}^{\gamma }}{qs\gamma }+\frac{q{y}^{\gamma }}{\gamma }+\frac{r{z}^{\gamma }}{\gamma }.$$

$${U}_{\text{31,32}}\left(x,y,z,t\right)=\pm \frac{i\sqrt{q}\sqrt{s}{\text{Coth}}\left[\frac{\psi }{2}\right]}{\sqrt{2}};\uppsi =-\frac{s{t}^{\gamma }}{\gamma }+\frac{2(r-s){x}^{\gamma }}{qs\gamma }+\frac{q{y}^{\gamma }}{\gamma }+\frac{r{z}^{\gamma }}{\gamma }.$$

$${U}_{\text{33,34}}\left(x,y,z,t\right)=\pm i\sqrt{2}\sqrt{q}\sqrt{s}{\text{Tanh}}\left[\psi \right];\uppsi =-\frac{s{t}^{\gamma }}{\gamma }+\frac{(r-s){x}^{\gamma }}{2qs\gamma }+\frac{q{y}^{\gamma }}{\gamma }+\frac{r{z}^{\gamma }}{\gamma }.$$

$${U}_{\text{35,36}}\left(x,y,z,t\right)=\pm \frac{i\sqrt{q}\sqrt{s}{\text{Tanh}}\left[\frac{\psi }{2}\right]}{\sqrt{2}};\uppsi =-\frac{s{t}^{\gamma }}{\gamma }+\frac{2(r-s){x}^{\gamma }}{qs\gamma }+\frac{q{y}^{\gamma }}{\gamma }+\frac{r{z}^{\gamma }}{\gamma }.$$

$${U}_{\text{37,38,39,40}}\left(x,y,z,t\right)=\pm \frac{\sqrt{q}\sqrt{s}\left({\text{Sech}}\left[\psi \right]\pm i{\text{Tanh}}\left[\psi \right]\right)}{\sqrt{2}};\uppsi =-\frac{s{t}^{\gamma }}{\gamma }+\frac{2(r-s){x}^{\gamma }}{qs\gamma }+\frac{q{y}^{\gamma }}{\gamma }+\frac{r{z}^{\gamma }}{\gamma }.$$

$${U}_{\text{41,42}}\left(x,y,z,t\right)=\pm i\sqrt{2}\sqrt{q}\sqrt{s}{\text{Csch}}[\psi ];\uppsi =-\frac{s{t}^{\gamma }}{\gamma }+\frac{(-r+s){x}^{\gamma }}{qs\gamma }+\frac{q{y}^{\gamma }}{\gamma }+\frac{r{z}^{\gamma }}{\gamma }.$$

$${U}_{\text{43,44,45,46}}\left(x,y,z,t\right)=\pm \frac{\sqrt{2}\sqrt{q}\sqrt{s}{\text{Sech}}[\psi ]}{\sqrt{2}\pm {\text{Tanh}}[\psi ]};\uppsi =-\frac{s{t}^{\gamma }}{\gamma }+\frac{(-r+s){x}^{\gamma }}{qs\gamma }+\frac{q{y}^{\gamma }}{\gamma }+\frac{r{z}^{\gamma }}{\gamma }.$$

$${U}_{\text{47,48}}\left(x,y,z,t\right)=\pm \frac{i\sqrt{2}\sqrt{q}\sqrt{s}{\text{Sech}}[\psi ]}{\sqrt{3}\pm 2{\text{Tanh}}[\psi ]};\uppsi =-\frac{s{t}^{\gamma }}{\gamma }+\frac{(-r+s){x}^{\gamma }}{qs\gamma }+\frac{q{y}^{\gamma }}{\gamma }+\frac{r{z}^{\gamma }}{\gamma }.$$

$${U}_{\text{49,50}}\left(x,y,z,t\right)=\pm \sqrt{2}\sqrt{q}\sqrt{s}{\text{Sech}}[\psi ];\uppsi =-\frac{s{t}^{\gamma }}{\gamma }+\frac{(-r+s){x}^{\gamma }}{qs\gamma }+\frac{q{y}^{\gamma }}{\gamma }+\frac{r{z}^{\gamma }}{\gamma }.$$

### The 3rd WBBM equation

The third WBBM equation has the following solution sets when the process mentioned above is followed:

$$s=-\frac{q}{-1+{p}^{2}}, {a}_{0}=0, {a}_{1}=\pm \frac{i\sqrt{2}p\sqrt{q}\sqrt{-{b}_{0}^{2}+{d}_{1}^{2}}}{\sqrt{r-{p}^{2}r}}, {b}_{1}=0, {c}_{1}=0.$$

$$s=\frac{2q}{2+{p}^{2}}, {a}_{0}=\pm \frac{pq{d}_{1}}{\frac{2\sqrt{q}r}{\sqrt{-(2+{p}^{2})r}}+\frac{{p}^{2}\sqrt{q}r}{\sqrt{-(2+{p}^{2})r}}}, {a}_{1}=\pm \frac{\sqrt{{p}^{2}q{b}_{0}^{2}-{p}^{2}q{d}_{1}^{2}}}{\sqrt{2r+{p}^{2}r}}, {b}_{1}=0, {c}_{1}=\pm \frac{p\sqrt{q}{b}_{0}}{\sqrt{-2r-{p}^{2}r}}.$$

$$s=\frac{2q}{2+{p}^{2}}, {a}_{0}=0, {a}_{1}=\pm \frac{\sqrt{{p}^{2}q{b}_{0}^{2}-{p}^{2}q{b}_{1}^{2}}}{\sqrt{2r+{p}^{2}r}}, {c}_{1}=\pm \frac{p\sqrt{q}{b}_{0}}{\sqrt{-2r-{p}^{2}r}}, {d}_{1}=0.$$

$$s=\frac{q}{1+2{p}^{2}}, {a}_{0}=\frac{2pq{d}_{1}}{\frac{\sqrt{2}\sqrt{q}r}{\sqrt{-(1+2{p}^{2})r}}+\frac{2\sqrt{2}{p}^{2}\sqrt{q}r}{\sqrt{-(1+2{p}^{2})r}}}, {a}_{1}=0, {b}_{1}=0, {c}_{1}=-\frac{\sqrt{2}p\sqrt{q}{b}_{0}}{\sqrt{-r-2{p}^{2}r}}.$$

$$s=\frac{q}{1+2{p}^{2}}, {a}_{0}=\frac{2pq{d}_{1}}{-\frac{\sqrt{2}\sqrt{q}r}{\sqrt{-(1+2{p}^{2})r}}-\frac{2\sqrt{2}{p}^{2}\sqrt{q}r}{\sqrt{-(1+2{p}^{2})r}}}, {a}_{1}=0, {b}_{1}=0, {c}_{1}=\frac{\sqrt{2}p\sqrt{q}{b}_{0}}{\sqrt{-r-2{p}^{2}r}}.$$

$$s=-\frac{q}{-1+{p}^{2}}, {a}_{0}=0, {a}_{1}=\pm \frac{\sqrt{2}p\sqrt{q}{d}_{1}}{\sqrt{-r+{p}^{2}r}}, {b}_{0}=0, {b}_{1}=0, {c}_{1}=0.$$

$$s=-\frac{q}{-1+{p}^{2}}, {a}_{0}=0, {a}_{1}=\pm \frac{\sqrt{2}p\sqrt{q}{d}_{1}}{\sqrt{r-{p}^{2}r}}, {b}_{0}=-\sqrt{2}{d}_{1}, {b}_{1}=0, {c}_{1}=0.$$

$$s=-\frac{q}{-1+{p}^{2}}, {a}_{0}=0, {a}_{1}=\pm \frac{\sqrt{2}p\sqrt{q}{d}_{1}}{\sqrt{r-{p}^{2}r}}, {b}_{0}=\sqrt{2}{d}_{1}, {b}_{1}=0, {c}_{1}=0.$$

$$s=-\frac{q}{-1+{p}^{2}}, {a}_{0}=0, {a}_{1}=\pm \frac{p\sqrt{q}{d}_{1}}{\sqrt{-2r-{p}^{2}r}}, {b}_{0}=\pm \frac{\sqrt{\frac{3}{2}} \sqrt{{d}_{1}^{2}+{p}^{2}{d}_{1}^{2}}}{\sqrt{2+{p}^{2}}}, {b}_{1}=0, {c}_{1}=0.$$

$$s=-\frac{q}{-1+{p}^{2}}, {a}_{0}=0, {a}_{1}=\pm \frac{\sqrt{2}p\sqrt{q}{b}_{0}}{\sqrt{r-{p}^{2}r}}, {b}_{1}=0, {c}_{1}=0, {d}_{1}=0.$$

$$s=\frac{2q}{2+{p}^{2}}, {a}_{0}=\pm \frac{p\sqrt{q}{d}_{1}}{\sqrt{-2r-{p}^{2}r}}, {a}_{1}=\pm \frac{p\sqrt{q}{d}_{1}}{\sqrt{-2r-{p}^{2}r}}, {b}_{0}=0, {b}_{1}=0, {c}_{1}=0.$$

$$s=\frac{2q}{2+{p}^{2}}, {a}_{0}=0, {a}_{1}=\pm \frac{p\sqrt{q}{b}_{0}}{\sqrt{2r+{p}^{2}r}}, {b}_{1}=0, {c}_{1}=\pm \frac{p\sqrt{q}{b}_{0}}{\sqrt{-2r-{p}^{2}r}}, {d}_{1}=0.$$

$$s=\frac{2q}{2+{p}^{2}}, {a}_{0}=0, {a}_{1}=0, {b}_{0}=\pm {b}_{1}, {c}_{1}=\pm \frac{p\sqrt{q}{b}_{1}}{\sqrt{-2r-{p}^{2}r}}, {d}_{1}=0.$$

$$s=\frac{q}{1+2{p}^{2}}, {a}_{0}=0, {a}_{1}=0, {b}_{1}=0, {c}_{1}=\pm \frac{\sqrt{2}p\sqrt{q}{b}_{0}}{\sqrt{-r-2{p}^{2}r}}, {d}_{1}=0.$$

Concerning these solution sets, we acquired the exact solutions of the equation

$${U}_{\text{51,52}}\left(x,y,z,t\right)=\pm \frac{i\sqrt{2}p\sqrt{q}{\text{Sech}}[\psi ]\sqrt{-{b}_{0}^{2}+{d}_{1}^{2}}}{\sqrt{r-{p}^{2}r}({b}_{0}+{d}_{1}{\text{Tanh}}[\psi ])};\uppsi =\frac{q{t}^{\gamma }}{(-1+{p}^{2})\gamma }+\frac{p{x}^{\gamma }}{\gamma }+\frac{q{y}^{\gamma }}{\gamma }+\frac{r{z}^{\gamma }}{\gamma }.$$

$${U}_{\text{53,54,55,56}}\left(x,y,z,t\right)=\pm \frac{p\sqrt{q}\sqrt{(2+{p}^{2})r}{d}_{1}\pm \sqrt{-(2+{p}^{2})r}{\text{Sech}}[\psi ]\sqrt{{p}^{2}q({b}_{0}^{2}-{d}_{1}^{2})}+p\sqrt{q}\sqrt{(2+{p}^{2})r}{b}_{0}{\text{Tanh}}[\psi ]}{\sqrt{-{\left(2+{p}^{2}\right)}^{2}{r}^{2}}\left({b}_{0}+{d}_{1}{\text{Tanh}}\left[\psi \right]\right)};\uppsi =-\frac{2q{t}^{\gamma }}{(2+{p}^{2})\gamma }+\frac{p{x}^{\gamma }}{\gamma }+\frac{q{y}^{\gamma }}{\gamma }+\frac{r{z}^{\gamma }}{\gamma }.$$

$${U}_{\text{57,58,59,60}}\left(x,y,z,t\right)=\pm \frac{\frac{{\text{Sech}}[\psi ]\sqrt{{p}^{2}q({b}_{0}^{2}-{b}_{1}^{2})}}{\sqrt{(2+{p}^{2})r}}\pm \frac{p\sqrt{q}{b}_{0}{\text{Tanh}}[\psi ]}{\sqrt{-(2+{p}^{2})r}}}{{b}_{0}+{\text{Sech}}[\psi ]{b}_{1}};\uppsi =-\frac{2q{t}^{\gamma }}{(2+{p}^{2})\gamma }+\frac{p{x}^{\gamma }}{\gamma }+\frac{q{y}^{\gamma }}{\gamma }+\frac{r{z}^{\gamma }}{\gamma }.$$

$${U}_{\text{61,62}}\left(x,y,z,t\right)=\pm \frac{\sqrt{2}p\sqrt{q}({d}_{1}+{b}_{0}{\text{Tanh}}[\psi ])}{\sqrt{-(1+2{p}^{2})r}({b}_{0}+{d}_{1}{\text{Tanh}}[\psi ])};\uppsi =-\frac{q{t}^{\gamma }}{(1+2{p}^{2})\gamma }+\frac{p{x}^{\gamma }}{\gamma }+\frac{q{y}^{\gamma }}{\gamma }+\frac{r{z}^{\gamma }}{\gamma }.$$

$${U}_{\text{63,64}}\left(x,y,z,t\right)=\pm \frac{\sqrt{2}p\sqrt{q}{\text{Csch}}[\psi ]}{\sqrt{(-1+{p}^{2})r}};\uppsi =\frac{q{t}^{\gamma }}{(-1+{p}^{2})\gamma }+\frac{p{x}^{\gamma }}{\gamma }+\frac{q{y}^{\gamma }}{\gamma }+\frac{r{z}^{\gamma }}{\gamma }.$$

$${U}_{\text{65,66}}\left(x,y,z,t\right)=\pm \frac{\sqrt{2}p\sqrt{q}{\text{Sech}}[\psi ]}{\sqrt{r-{p}^{2}r}\left(\sqrt{2}-{\text{Tanh}}\left[\psi \right]\right)};\uppsi =\frac{q{t}^{\gamma }}{(-1+{p}^{2})\gamma }+\frac{p{x}^{\gamma }}{\gamma }+\frac{q{y}^{\gamma }}{\gamma }+\frac{r{z}^{\gamma }}{\gamma }.$$

$${U}_{\text{67,68}}\left(x,y,z,t\right)=\pm \frac{\sqrt{2}p\sqrt{q}{\text{Sech}}[\psi ]}{\sqrt{r-{p}^{2}r}(\sqrt{2}+{\text{Tanh}}[\psi ])};\uppsi =\frac{q{t}^{\gamma }}{(-1+{p}^{2})\gamma }+\frac{p{x}^{\gamma }}{\gamma }+\frac{q{y}^{\gamma }}{\gamma }+\frac{r{z}^{\gamma }}{\gamma }.$$

$${U}_{\text{69,70,71,72}}\left(x,y,z,t\right)=\pm \frac{2p\sqrt{2+{p}^{2}}\sqrt{q}{d}_{1}}{\sqrt{-\left(2+{p}^{2}\right)r}\left(\sqrt{6}{\text{Cosh}}\left[\psi \right]\sqrt{\left(1+{p}^{2}\right){d}_{1}^{2}}\pm 2\sqrt{2+{p}^{2}}{\text{Sinh}}\left[\psi \right]{d}_{1}\right)};\uppsi =\frac{q{t}^{\gamma }}{(-1+{p}^{2})\gamma }+\frac{p{x}^{\gamma }}{\gamma }+\frac{q{y}^{\gamma }}{\gamma }+\frac{r{z}^{\gamma }}{\gamma }.$$

$${U}_{\text{73,74}}\left(x,y,z,t\right)=\pm \frac{\sqrt{2}p\sqrt{q}{\text{Sech}}\left[\psi \right]}{\sqrt{r-{p}^{2}r}};\uppsi =\frac{q{t}^{\gamma }}{(-1+{p}^{2})\gamma }+\frac{p{x}^{\gamma }}{\gamma }+\frac{q{y}^{\gamma }}{\gamma }+\frac{r{z}^{\gamma }}{\gamma }.$$

$${U}_{\text{75,76}}\left(x,y,z,t\right)=\pm \frac{p\sqrt{q}{\text{Coth}}\left[\frac{\psi }{2}\right]}{\sqrt{-(2+{p}^{2})r}};\uppsi =-\frac{2q{t}^{\gamma }}{(2+{p}^{2})\gamma }+\frac{p{x}^{\gamma }}{\gamma }+\frac{q{y}^{\gamma }}{\gamma }+\frac{r{z}^{\gamma }}{\gamma }.$$

$${U}_{\text{77,78}}\left(x,y,z,t\right)=\pm \frac{p\sqrt{q}{\text{Tanh}}\left[\frac{\psi }{2}\right]}{\sqrt{-(2+{p}^{2})r}};\uppsi =-\frac{2q{t}^{\gamma }}{(2+{p}^{2})\gamma }+\frac{p{x}^{\gamma }}{\gamma }+\frac{q{y}^{\gamma }}{\gamma }+\frac{r{z}^{\gamma }}{\gamma }.$$

$${U}_{\text{79,80,81,82}}\left(x,y,z,t\right)=\pm \frac{p\sqrt{q}{\text{Sech}}\left[\psi \right]\left(\sqrt{-\left(2+{p}^{2}\right)r}\pm \sqrt{\left(2+{p}^{2}\right)r}{\text{Sinh}}\left[\psi \right]\right)}{\sqrt{-{(2+{p}^{2})}^{2}{r}^{2}}};\uppsi =-\frac{2q{t}^{\gamma }}{(2+{p}^{2})\gamma }+\frac{p{x}^{\gamma }}{\gamma }+\frac{q{y}^{\gamma }}{\gamma }+\frac{r{z}^{\gamma }}{\gamma }.$$

$${U}_{\text{83,84}}\left(x,y,z,t\right)=-\frac{\sqrt{2}p\sqrt{q}{\text{Tanh}}\left[\psi \right]}{\sqrt{-(1+2{p}^{2})r}};\uppsi =-\frac{q{t}^{\gamma }}{(1+2{p}^{2})\gamma }+\frac{p{x}^{\gamma }}{\gamma }+\frac{q{y}^{\gamma }}{\gamma }+\frac{r{z}^{\gamma }}{\gamma }.$$

## Result and discussion

**Figure 2:**
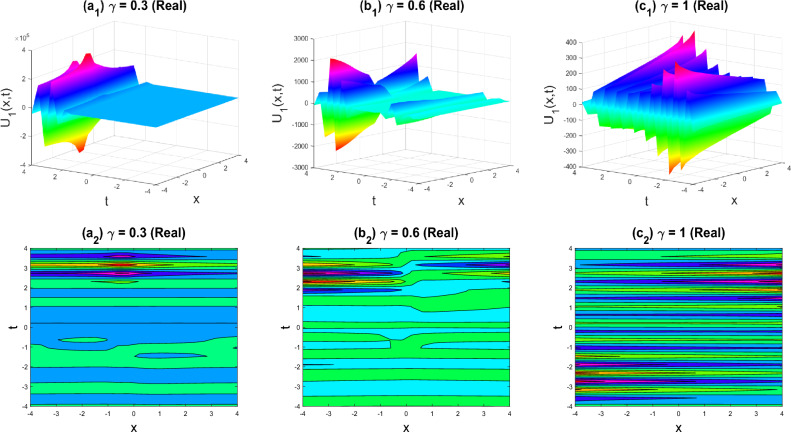
Multiple soliton shapes of the real part of ${U}_{1}\left(x,y,z,t\right)$ for the parameters $q=0.5, r=-0.5, s=0.5, {b}_{0}=1, {b}_{1}=0.1, y=0, z=0, \gamma =0.3, 0.6, 1$.

**Figure 3:**
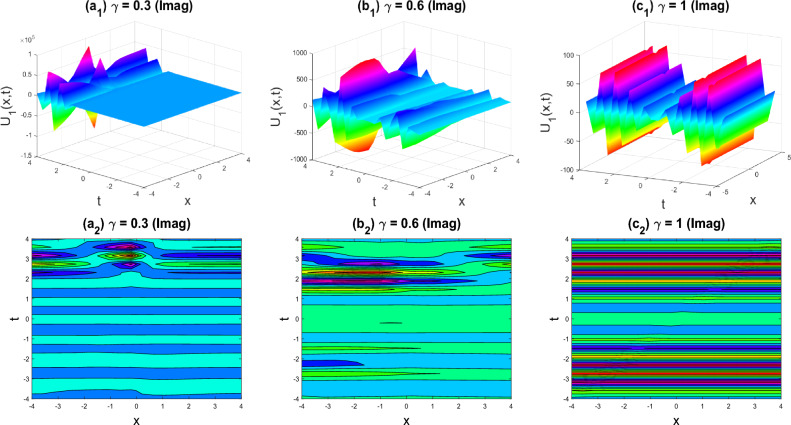
Multiple Kink-soliton shapes of the imaginary part of ${U}_{1}\left(x,y,z,t\right)$ for the parameters $q=0.5, r=-0.5, s=0.5, {b}_{0}=1, {b}_{1}=0.1, y=0, z=0, \gamma =0.3, 0.6, 1$.

**Figure 4:**
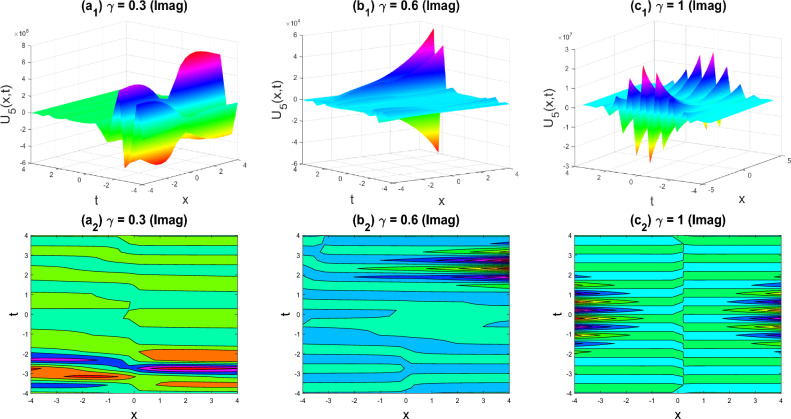
The breather-kink shape of the imaginary part of ${U}_{5}\left(x,y,z,t\right)$ for the parameters $q=0.5, r=-0.5, s=0.5, {b}_{0}=1, {d}_{1}=0.1, y=0, z=0, \gamma =0.3, 0.6, 1$.

**Figure 5:**
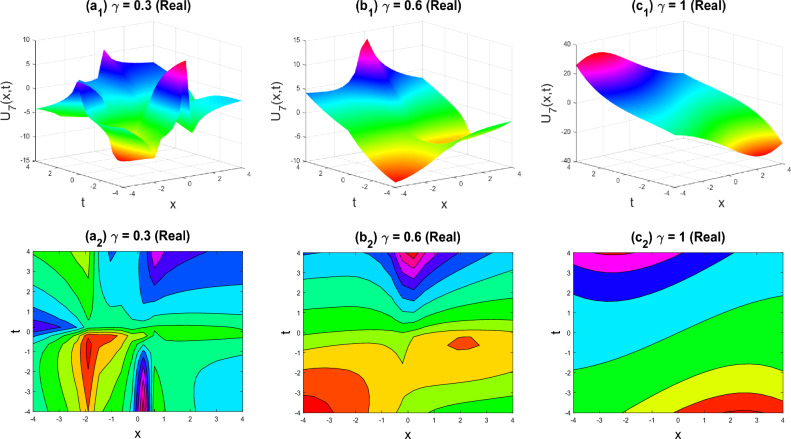
Kink-soliton shape of the real part of ${U}_{7}\left(x,y,z,t\right)$ for the parameters $q=0.5, r=-0.5, s=0.5, y=0, z=0, \gamma =0.3, 0.6, 1$.

**Figure 6:**
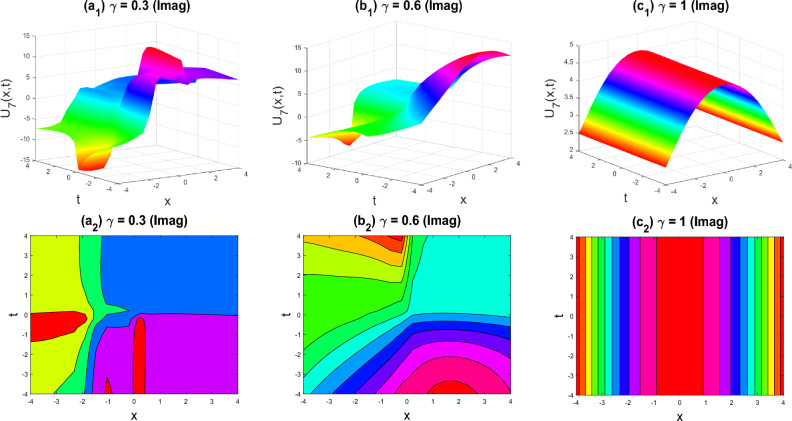
Soliton shape of the imaginary part of ${U}_{7}\left(x,y,z,t\right)$ for the parameters $q=0.5, r=-0.5, s=0.5, y=0, z=0, \gamma =0.3, 0.6, 1$.

**Figure 7:**
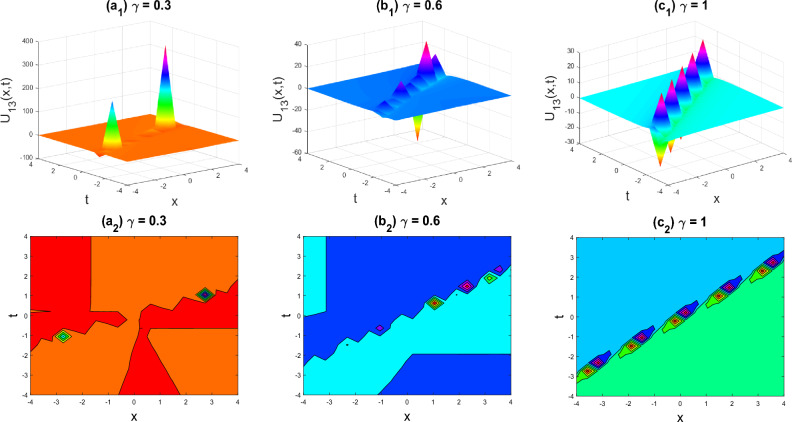
Multiple soliton shapes of the imaginary part of ${U}_{13}\left(x,y,z,t\right)$ for the parameters $q=0.5, r=-0.5, s=0.5, y=0, z=0, \gamma =0.3, 0.6, 1$.

**Figure 8:**
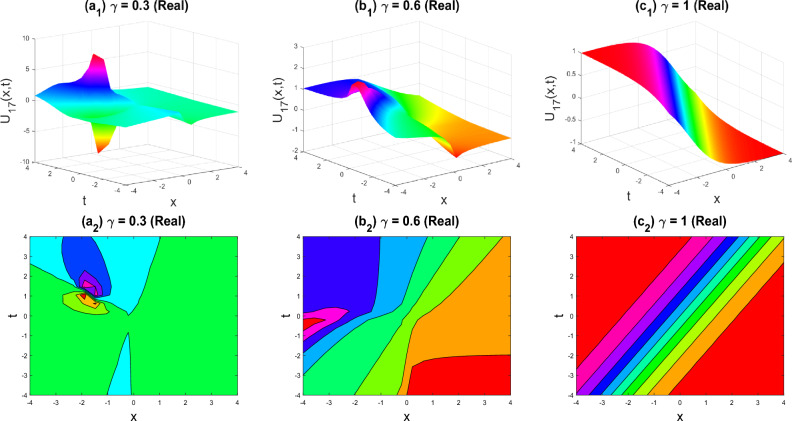
The lump-kink shape of the imaginary part of ${U}_{17}\left(x,y,z,t\right)$ for the parameters $q=0.5, r=-0.5, s=0.5, y=0, z=0, \gamma =0.3, 0.6, 1$.

**Figure 9:**
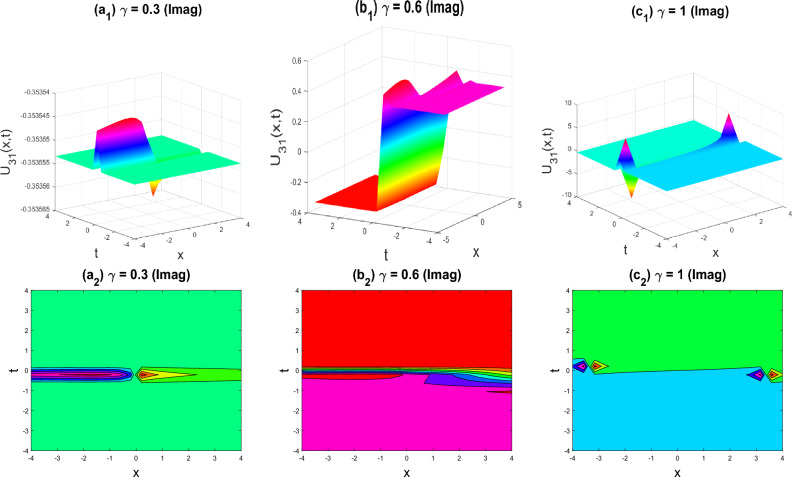
Kink-double soliton shape of the imaginary part of ${U}_{31}\left(x,y,z,t\right)$ for the parameters $q=0.5, r=-0.5, s=0.5, y=0, z=0, \gamma =0.3, 0.6, 1$.

**Figure 10:**
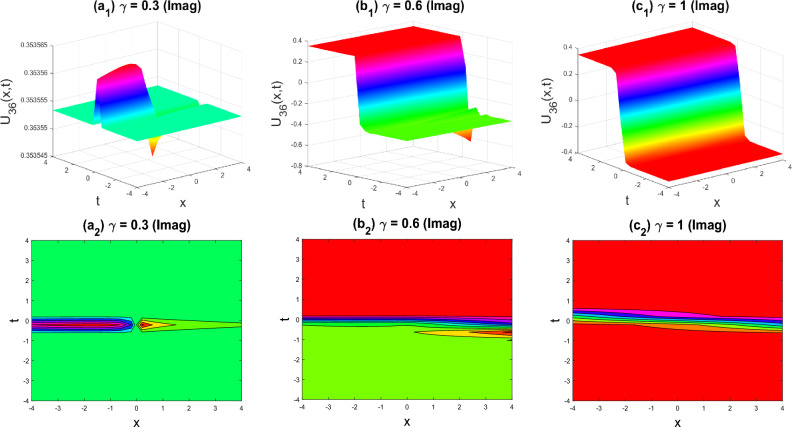
Kink shape of the imaginary part of ${U}_{36}\left(x,y,z,t\right)$ for the parameters $q=0.5, r=-0.5, s=0.5, y=0, z=0, \gamma =0.3, 0.6, 1$.

**Figure 11:**
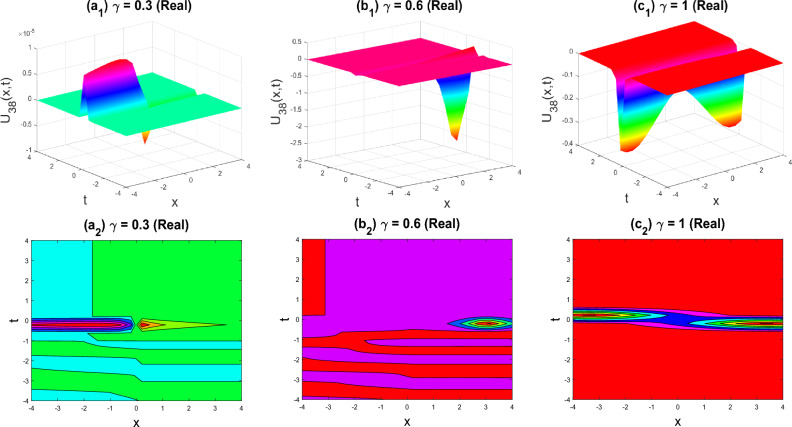
The cusp shape of the imaginary part of ${U}_{38}\left(x,y,z,t\right)$ for the parameters $q=0.5, r=-0.5, s=0.5, y=0, z=0, \gamma =0.3, 0.6, 1$.

**Figure 12:**
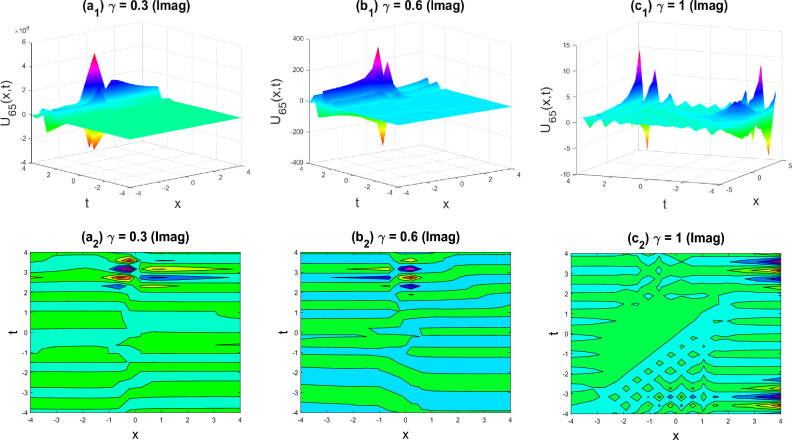
Kink-soliton shape of the imaginary part of ${U}_{65}\left(x,y,z,t\right)$ for the parameters $p=0.5, q=0.5, r=-0.5, y=0, z=0, \gamma =0.3, 0.6, 1$.

**Figure 13:**
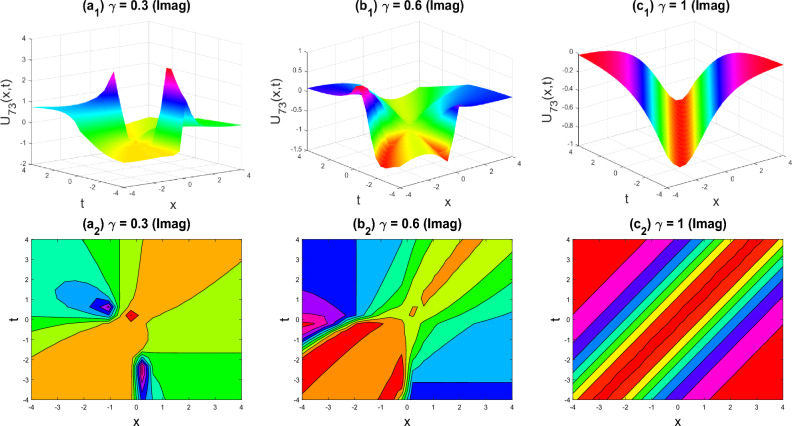
Dark-soliton shape of the imaginary part of ${U}_{73}\left(x,y,z,t\right)$ for the parameters $p=0.5, q=0.5, r=-0.5, y=0, z=0, \gamma =0.3, 0.6, 1$.

In this section, recently identified exact solutions to the family of 3D fractional WBBM equations are defined using physical and pictorial examples. Visualization is the ideal method for illustrating the entire crucial structure of real-life situations. Additionally, we took advantage of the computational bundle MATLAB capabilities by choosing appropriate fractional values of $\gamma$. We charged several exorbitant fees for the unknown aspects to evaluate its standard attributes. Figures 2, 3, 4, 5, 6, 7, 8, 9, 10, 11, 12 and 13 show the proven equations in detail.

### Physical explanation

A travelling wave that develops on the surface of an ocean, lake, or river is an iconic illustration of this type of wave. Gravity and surface tension forces work together to control the velocity of water waves, which results in their behavior. Imagine a tranquil body of water at rest—moving waveforms when there is an obstruction on the water's surface. Numerous things might contribute to this disturbance, such as the wind blowing over the water, a foreign object being dumped, and seismic activity. Let's take the example of the wind creating pond ripples. Surface, or surface tension, is a cohesive force that pulls water molecules together at a body of water's surface. Gravity is also pushing the water downhill at the same time.

When these two forces interact, restoring power is produced that tends to return the water's surface to its equilibrium position. As the wind blows over the water, it transfers energy to the surface, forcing some water molecules to flow upward to form peaks known as crests and downward to create troughs. The movement of water molecules up and down causes oscillations all over the water's surface, which is how the disturbance spreads. Water molecules close to one another interact, exchanging momentum and energy. These oscillations are spread throughout the water by the cohesive forces (surface tension) and gravity in conjunction with slight horizontal water molecule displacement. The wave's wavelength and water depth are two variables that affect the pace at which a water wave moves. The acceleration caused by gravity and the depth of the water are the main factors that affect the wave speed of deep-water waves. The movement of the water wave moves energy from one area to another. Without moving a sizable quantity of water mass in the direction of propagation, the wave spreads the significance of the original disturbance over the water's surface. Water waves may interact with other waves and cause interference patterns. This is known as dissipation. While destructive interference can result in wave cancellation, constructive interference increases wave amplitudes, resulting in more giant waves. Long-distance water waves eventually lose energy due to friction and viscosity as they move through the water, which causes the waves to dissipate. Water waves are essential in coastal dynamics, maritime transportation, and engineering. Foreseeing coastal erosion, constructing maritime constructions, and ensuring marine safety depend on understanding how ocean waves behave.

### Graphical explanation

The various forms of the 3D fractional WBBM equation solution are represented in Figs. 2, 3, 4, 5, 6, 7, 8, 9, 10, 11, 12 and 13. For each figure, the first row stands for the 3D surface plot, and the second stands for the contour plot. Here, we displayed the contour and 3D surface plots of many solutions. We have established some new solutions: kink shape, multiple kink shape, soliton shape, singular soliton shape, multiple soliton shape, dark soliton shape, double soliton shape, lump shape, etc. All the figures have been changed for the different values of $\gamma , (0.3\le \gamma \le 1)$. For changing the value of $\gamma$ from $0.3$ to $1$, sometimes the solution shapes turn into singular soliton shapes to multiple soliton shapes, singular kink-soliton shapes to multiple kink-soliton shapes, soliton shapes to dark soliton shapes, and so on. The nonlinearity of the wave causes its profile to vary over time as it passes through the medium, but dispersion balances this change and keeps the wave from spreading out or losing its form. In sum, solitary waves are fascinating phenomena that result from a careful balancing act between dispersion and nonlinearity in a medium. These waves are essential in many branches of physics and engineering because of their ability to keep their form and travel great distances without dispersing or losing energy, thanks to this equilibrium.

## Conclusion

Using the rational sine-Gordon expansion method, we identified the travelling wave solution for the 3D fractional WBBM issues family. The rational sine-Gordon expansion is a well-proven method for solving nonlinear partial differential equations. Numerous illustrated solitons, including singular, singular kink, periodic, lump, and asymptotic type solitons built using exponential, hyperbolic, and trigonometric structures, may be seen when the wave profile is examined for the generated generic parametric values. We have notably emphasized how the values or quantities of changes for different values of only one parameter ($\gamma$) impact the dynamic behavior of the water waves. Exponential and trigonometric functions are used to express the determined solutions. The physical meaning of the travelling wave solutions in this study will be explained by the space–time fractional NLS+, NLS−, and UNLS models from nonlinear optics, fluid mechanics, quantum theory, and other theoretical and numerical disciplines. This approach applies to intricate nonlinear physics, engineering, and applied mathematics models. It's possible that not every kind of nonlinear problem can be solved using this technique. It may not be able to solve solutions for more broad or intricate nonlinear systems, but it functions effectively for some particular types of equations. Use alternate techniques to validate the derived answers, such as asymptotic analysis, numerical simulations, or, if available, comparison with experimental data. This contributes to ensuring the dependability and correctness of the produced solutions. As a component of a more comprehensive toolset, apply the logical Sine-Gordon expansion approach. Combine it with additional perturbation, analytical, or numerical techniques to counterbalance its shortcomings and strengthen its points.

## Data Availability

The data used to support the findings of this study are available from the corresponding author upon request.
